# Granulomatous mastitis: from localized inflammation to systemic immune-mediated disorder

**DOI:** 10.3389/fmed.2026.1747120

**Published:** 2026-02-27

**Authors:** Yingying Dong, Qi Wang, Mengning Zhang, Lujia Zhang, Yan Liu, Tiantian Lei, Hong Zhao

**Affiliations:** 1 The First School of Clinical Medicine, Zhejiang Chinese Medical University, Hangzhou, China; 2 Department of Breast Surgery, Jiaxing Hospital of Traditional Chinese Medicine, Jiaxing, China; 3 Department of Nephrology, The First Affiliated Hospital of Zhejiang Chinese Medical University (Zhejiang Provincial Hospital of Chinese Medicine), Hangzhou, China; 4 Zhejiang Key Laboratory of Research and Translation for Kidney Deficiency-Stasis-Turbidity Disease, Hangzhou, China; 5 Department of Breast Surgery, The First Affiliated Hospital of Zhejiang University of Traditional Chinese Medicine (Zhejiang Provincial Hospital of Traditional Chinese Medicine), Hangzhou, China

**Keywords:** breast, breast inflammation, granulomatous mastitis, immune system, prolactin, traditional Chinese medicine

## Abstract

Granulomatous mastitis (GM) is a chronic inflammatory breast disease of unknown etiology, characterized by a high recurrence rate and challenging clinical management. This review reconceptualizes GM as an immune-mediated disorder and delineates the aberrant crosstalk between innate and adaptive immunity that constitutes its core pathogenesis. We emphasize that hyperprolactinemia acts as a pivotal driver, initiating a pro-inflammatory cascade characterized by macrophage M1 polarization, neutrophil extracellular trap (NET) release, NK cell activation, and severe disruption of T and B cell homeostasis. Currently, glucocorticoids (GCs) are routinely used in the treatment of Granulomatous mastitis, but their efficacy is limited and they cannot fulfill all the needs of clinical treatment. Therefore, it has become imperative to adopt immunomodulatory strategies for treatment. By synthesizing the evolving understanding of GM’s immunopathology, this review aims to bridge the gap between mechanistic insights and clinical practice. We critically assess current and emerging therapeutics, including the potential role of Traditional Chinese Medicine (TCM), and propose a framework for future targeted therapeutic strategies that modulate specific immune pathways in GM.

## 1 Introduction

Granulomatous mastitis (GM) is a benign inflammatory disease that occurs in the breast and usually has a long course. It is typically characterized by the formation of nonspecific granulomas in the breast tissue (1, 2). Patients often present with breast lumps, abscesses, localized pain, redness of the skin, and even fistulas and scarring (3, 4). The exact cause of the disease is still unclear and controversial, so many cases are defined as “idiopathic,” meaning that the cause is unknown (5).

The nomenclature for this condition has not yet been standardized globally and is typically determined based on its etiology and histopathological characteristics. Common terms in the literature include granulomatous mastitis (GM) (6), granulomatous lobular mastitis (GLM) (7), idiopathic granulomatous mastitis (IGM) (8), and idiopathic granulomatous lobular mastitis (IGLM) (3). Cystic neutrophilic granulomatous mastitis (CNGM) represents a rare subtype often associated with *Bacillus cereus* infection (9, 10). Although GM is the standard MeSH term, IGM is more frequently used in academic literature, and GLM is also commonly employed. This whole confusion in naming really comes down to experts not agreeing on what causes the disease. With more and more cases being reported worldwide, it’s really important to study it further. To maintain consistency in terminology throughout this paper, the abbreviation “GM” will be uniformly adopted in the following text.

Treatment of GM emphasizes individualization, with observation and preferred pharmacological treatment (e.g., antibiotics, corticosteroids, etc.) for mild cases and surgery for cases where medications are ineffective or accompanied by complications. In certain cases, traditional Chinese medicine therapies may be incorporated as adjunctive treatments. Treatment with oral corticosteroids usually lasts at least 2 to 3 months and may cause significant side effects. When local surgical excision is performed, the disease can come back in up to 50% of patients. Recurrence rates with systemic corticosteroids range from 16 to 50% (11). Because the cause of GM is not clear, its progression is often hard to predict. Repeated relapses remain a major challenge in clinical practice. In addition, GM can appear in different forms and its imaging features resemble those of other breast diseases, including breast cancer. This similarity sometimes leads to misdiagnosis or unnecessary medical procedures (12).

## 2 Epidemiological patterns and affected demographics

Although the incidence of GM is not significantly high globally, it has shown a noticeable upward trend over the past 20 years (2). Literature reports indicate that the annual incidence rate of GM is about 0.37%, with non-lactational mastitis occurring in approximately 3% of benign breast disease biopsies, accounting for 24% of all inflammatory breast diseases (13, 14). However, due to the passage of time, evolving understanding of the disease, and advancements in diagnostics, these figures may not accurately reflect the current real-world incidence.

While reports of GM are found worldwide, studies suggest that the prevalence of GM correlates with race, ethnicity, and geographical region. GM commonly occurs in the Mediterranean, developing countries in Asia, and the Middle East (Egypt, Turkey, Iran), with a higher incidence among Hispanic populations and Southeast Asians than among Caucasians (UK, USA, New Zealand) (13, 15, 16), and is more prevalent among non-white women.

In the United States, the estimated incidence rate is 2.4 per 100,000 women, which equates to 0.24%. A study by Óscar A. and colleagues, covering the period from January 1, 2016, to December 31, 2019, treated 848 patients with inflammatory breast diseases, 18 of whom were diagnosed with GM, resulting in an incidence rate of 2.12%, all among mixed-race populations (17). Research by Nina Capiro and others has shown that in the U.S., GM patients are more likely to list Spanish as their preferred language, identify as Hispanic/Latino, and be born in Mexico. Patients whose preferred language is Spanish are four times more likely to have GM compared to those whose preferred language is English. GM patients are also less likely to have a designated primary care provider, indicating potential difficulties in accessing healthcare. A 2009 CDC report further noted that all GM patients had an educational level lower than the sixth grade (18). This suggests that the occurrence of GM intertwines with certain social factors, possibly related to socioeconomic conditions and work stress, and provides insights for further exploration of the high incidence rates in developing countries in Asia.

Due to the differences in incidence rates across regions, there are relatively more cohort studies on GM in Asia and the Middle East (13, 19, 20). For instance, a systematic review by Martinez-Ramos et al. (21) included 70 retrospective studies, with Turkey contributing approximately 30% of the total number of studies. However, there is currently no report on the global incidence of this disease, and there remains a lack of worldwide studies on population differences regarding this condition. Additionally, the research by David Martinez-Ramos and others grouped countries based on the Human Development Index and concluded that there are some differences in clinical manifestations and treatment approaches between developed and developing countries. Palpable breast lumps are the most common clinical presentation in GM in developed countries, whereas pain is the most prevalent symptom in developing countries. Antibiotic treatment is most commonly used in developing countries, whereas surgical interventions are more frequent in developed countries.

Therefore, the incidence, clinical manifestations, and treatment of GM vary regionally. The correlation between GM incidence and ethnicity requires further large-scale, multifactorial analysis that considers a range of socio-economic factors, to explore the underlying associations and provide a theoretical basis for the prevention and control of this disease at the societal level.

## 3 Etiology and risk factors

GM is a rare non-cancerous inflammatory disease of the breast. It most often occurs in women during their reproductive years (22). The exact cause of GM is still not known. There is also limited strong evidence available to help with diagnosis and treatment. However, recent studies indicate that several complicated processes and factors are involved. At present, the main theories about what causes GM include autoimmune reactions, infections, and hormonal imbalances (11, 23).

Through a combination of diverse immune cells and antibody-related responses, the body drives persistent breast inflammation and granuloma formation (23). Studies show that GM patients generally have lower levels of Th cells compared to healthy individuals. At the same time, the number of cytotoxic T cells, natural killer (NK) cells and natural killer T (NKT) cells was generally increased. This phenomenon reveals the presence of systemic immune disorders in GM and provides evidence for autoimmune mechanisms (24). Immunohistochemical further confirmed the immune-mediated mechanism. Deng et al. (25) found that the diseased tissues not only had high levels of CD3+, CD4+, and CD8 + T cells, but also showed positive staining for the B-cell marker, CD79a, which indicated the potential roles of cellular and humoral immunity in the disease, respectively (2). Histologic comparisons revealed significantly higher numbers of plasma cells in GM tissues than in tuberculous mastitis (TM) samples. This finding provides direct histologic evidence for the role of humoral immune response in GM (23).

Multiple studies and case reports point to a connection between GM and autoimmune reactions. Back in 1977, Cohen (26) were the first to suggest that GM might actually be a localized expression of an autoimmune condition. We know that certain infections and diseases—like tuberculosis, sarcoidosis, fungal infections, and autoimmune disorders such as granulomatosis with polyangiitis and giant cell arteritis—can lead to GM. This range of causes also hints that GM onset could be tied to problems with the immune system. Importantly, GM does not only show up as breast inflammation. It can also present with symptoms outside the mammary gland, such as inflammatory arthritis, joint pain, uveitis, or erythema nodosum. These kinds of extramammary signs reinforce the idea that GM might be part of a broader, systemic immune process. On top of that, the fact that GM often responds well to corticosteroid treatment adds further support to the view that it could be an autoimmune disease (2). The effectiveness of corticosteroid treatment in many GM patients supports the view that immune dysregulation is central to this disease. Corticosteroids are potent anti-inflammatory agents widely used against autoimmune disorders (2, 27).

Hormonal factors are also believed to be involved in GM. Altered levels of estrogen and progesterone may directly influence its development. For example, oral contraceptive use or natural hormonal changes during pregnancy and breastfeeding can affect the breast ducts. These changes can cause the ducts to widen and fill with fluid, which may then damage the duct lining and trigger local inflammation (22, 28, 29). Other health conditions have also been associated with GM. These include physical breast injury, hyperprolactinemia (HPRL), sarcoidosis, tuberculosis, diabetes, alpha-1-antitrypsin deficiency, smoking, obesity, diet, and genetic predisposition. Certain medical conditions and medications may also play a role. For example, pituitary adenomas and some psychiatric drugs—such as risperidone and antidepressants like fluoxetine—can raise prolactin levels. This increase may then indirectly contribute to the onset of GM (2, 13).

## 4 Core immunopathological mechanisms

As discussed earlier, the development of GM is closely linked to several risk factors. These include autoimmune dysregulation and HPRL. However, how these different factors work together to produce the typical non-caseating granulomas—the main pathological feature of GM—within the breast tissue is still not fully understood (24).

### 4.1 Initiation: microenvironmental changes and danger signal emission

GM is initiated by alterations in the local breast microenvironment. Factors such as nipple retraction, HPRL, local trauma, surgery, or infection may act independently or in concert to cause ductal obstruction and subsequent milk stasis (23, 30). When milk remains stagnant in the ducts for an extended period, it can lead to ductal obstruction and increased intraluminal pressure. This pressure progressively damages the ductal wall, ultimately resulting in ductal rupture. Consequently, lipid components and other degraded milk constituents may extravasate into the adjacent breast tissue (31).

When these components extravasate from the damaged ducts into the breast tissue, they are displaced from their normal anatomical compartment. The breast parenchyma then recognizes these degraded milk substances as “damage-associated molecular patterns”(DAMPs) (32). The release of these endogenous DAMPs from dead or damaged cells activates innate immune cells and pro-inflammatory signaling, thus launching the local immune response (33, 34).

Notably, HPRL plays a dual role in this phase: it not only creates conditions for mechanical obstruction by promoting milk stasis but also, as an immunomodulatory hormone, may pre-condition the immune cell landscape within the breast tissue, thereby priming it for subsequent exaggerated inflammatory responses (23, 35).

### 4.2 Effector stage: from innate activation to adaptive launching

**Figure 1:**
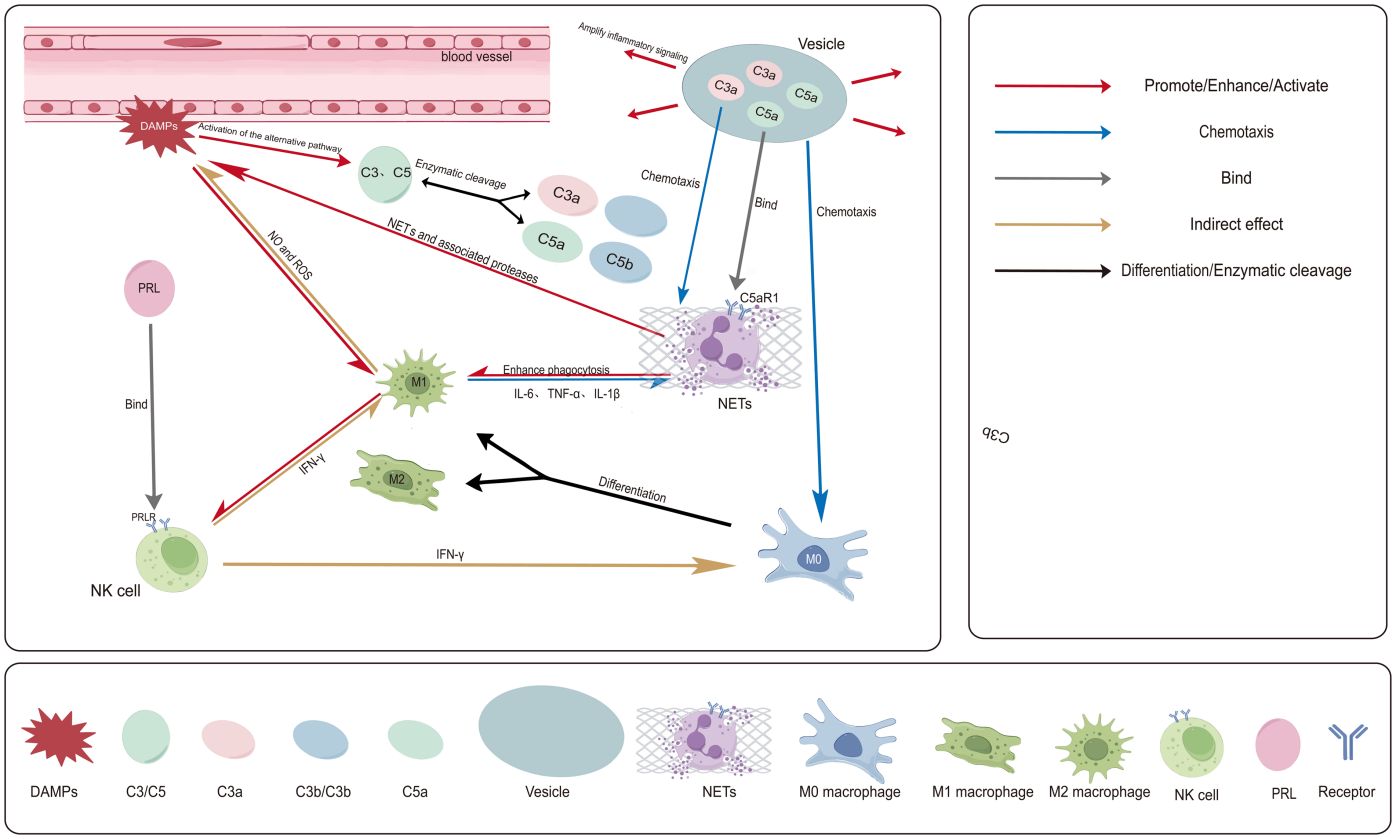
Initiating phase and innate immune activation in GM. Ductal rupture leads to the release of DAMPs, which activates the complement alternative pathway, generating C3a and C5a. These anaphylatoxins can be delivered via exosomes to amplify signaling. C5a potently recruits and activates neutrophils via its receptor C5aR1, promoting NETs release and exacerbating tissue damage. C5a/C3a also activates macrophages, which, driven by IFN-γ from sources such as NK cells, polarize into a pro-inflammatory M1 phenotype. M1 macrophages secrete IL-1β, IL-6, and TNF-α, further recruiting neutrophils and releasing toxic mediators like ROS that directly damage tissue. HPRL activates NK cells by binding to its receptor; the IFN-γ secreted by these cells drives M1 polarization, while the inflammatory milieu generated by M1 cells can in turn enhance NK cell activity, forming a positive feedback loop that exacerbates early inflammation. Persistent tissue injury leads to the release of new DAMPs, maintaining and amplifying this inflammatory cascade. Created with Figdraw.com.

As shown in Figure 1, following their release, DAMPs rapidly activate pro-inflammatory signaling cascades (33). Neutrophils, as the earliest and most abundantly recruited innate immune cells, are subsequently chemotactically drawn to the site of injury (36). They capture and eliminate pathogens through degranulation, phagocytosis, and the release of neutrophil extracellular traps (NETs) (37). Although NETs serve as a defensive mechanism against pathogens, their excessive or dysregulated release within the chronic inflammatory milieu of GM can instead exacerbate tissue damage. Furthermore, through mechanisms such as the exposure of autoantigens, NETs may initiate and amplify subsequent autoimmune responses. Aberrant NETs formation has now been implicated in a variety of autoimmune diseases (38, 39).

Subsequently, macrophages emerge as the central players on the inflammatory stage of GM. These cells represent the most widely distributed immune cell population within human tissues (40). Within GM lesions, they are recruited by local pro-inflammatory signals and undergo phenotypic and morphological differentiation, a process known as macrophage polarization (41). In the specific microenvironment of GM, IFN-*γ* produced by activated Th1 cells synergizes with DAMPs to collectively drive macrophage activation predominantly towards the M1 phenotype. M1 macrophages adopt a pro-inflammatory functional state characterized by potent phagocytic activity and cytotoxicity (42). They secrete large quantities of key pro-inflammatory cytokines such as IL-6 and IL-1β, amplifying both systemic and local inflammatory responses. This further recruits additional immune cells, thereby establishing a vicious cycle (43). Concurrently, M1 macrophages directly attack and induce damage and necrosis in breast tissue cells through the release of toxic mediators, such as reactive oxygen species and substantial amounts of nitric oxide. This represents the central and direct mechanism underlying the ongoing tissue destruction in GM (44, 45).

NK cells constitute another essential component of the innate immune system. They participate in immunomodulation through cytotoxicity and cytokine secretion. In GM, their activity is likely modulated by the distinct local microenvironment. It is established that NK cells express prolactin (PRL) receptors, and a subset of GM patients presents with elevated serum PRL levels (35). Relevant basic research indicates that PRL can directly enhance NK cell activation and cytotoxicity by upregulating the autocrine IL-2/IL-2Rα and IL-15/IL-15Rα pathways (46, 47). Therefore, given the inflammatory context of GM, locally elevated PRL levels likely exert a similar activating effect on NK cells. Activated NK cells secrete copious amounts of IFN-*γ*, which is a key factor driving macrophage polarization toward the pro-inflammatory M1 phenotype (48). Based on this, a theoretical model can be constructed: a positive feedback loop potentially exists among PRL, activated NK cells, and M1 macrophages—where PRL activates NK cells, which induce M1 macrophages via IFN-*γ*, and the inflammatory milieu generated by M1 macrophages may in turn further stimulate NK cells. This hypothetical model may provide novel insights into explaining the rapid escalation of the initial inflammatory response in GM.

Furthermore, certain DAMPs can directly activate the complement alternative pathway. Clinical studies have confirmed significantly elevated levels of the intrinsic complement components C3 and C4 in both the serum and lesion tissues of GM patients, which markedly decrease following treatment, suggesting a close association with disease activity (35, 49). C3 and C5 are components of the complement system and can be cleaved by proteases via the classical, alternative, or lectin pathways, generating the potent inflammatory mediators known as anaphylatoxins C3a and C5a. These anaphylatoxins bind to their specific receptors, C3aR and C5aR1, respectively, thereby inducing histamine release from mast cells and basophils and potently recruiting polymorphonuclear leukocytes—such as neutrophils and eosinophils—to the site of inflammation (50). Further research revealed that exosomes derived from GM lesions are highly enriched with C3a and C5a. Concurrently, the expression of the C3/C3a-C3aR and C5/C5a-C5aR1 signaling axes is synchronously upregulated. These findings suggest that the complement system further amplifies and sustains inflammatory signaling in a spatial manner through this “vesicle-mediated delivery” mechanism (51).

### 4.3 Sustained phase: adaptive immune dysregulation and granuloma development

#### 4.3.1 Polarization imbalance of T lymphocytes

**Figure 2:**
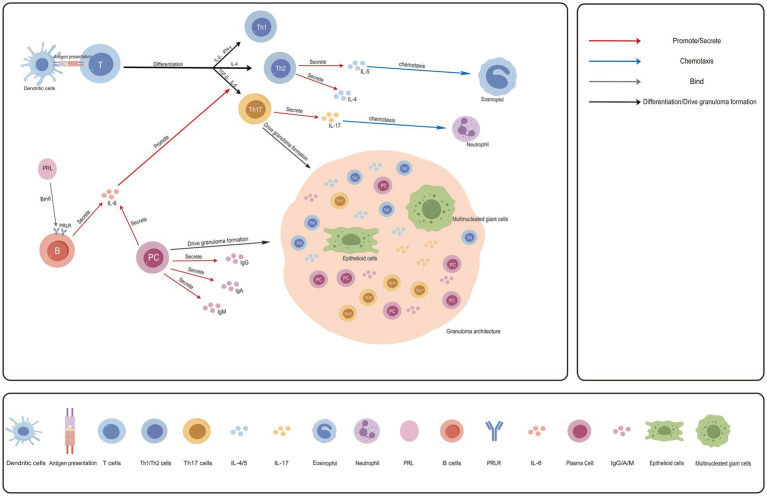
Adaptive immune dysregulation and disease chronicity in GM. The pathological cascade builds upon the inflammatory microenvironment established by innate immunity. Dendritic cells present autoantigens to activate naive T cells. Driven by IL-4, these T cells polarize toward a Th2 phenotype. Th2 cells then secrete IL-5, which recruits and activates eosinophils. Concurrently, an aberrantly amplified Th17 response, primarily driven by IL-6 from sources including B cells along with TGF-β, robustly and persistently recruits neutrophils through the secretion of IL-17, leading to direct tissue destruction. B cells, whose activation is facilitated by hyperprolactinemia, differentiate into plasma cells with help from Th2 cells. These plasma cells produce autoantibodies such as IgA, IgM, and IgG, and also secrete IL-6. This IL-6 further promotes Th17 differentiation, thereby creating a self-reinforcing positive feedback loop that perpetuates chronic inflammation. This entire process occurs alongside a significant functional impairment of both Treg cells and Breg cells. This regulatory failure renders them unable to effectively suppress the aberrant immune responses described, ultimately culminating in the formation of granulomatous structures and the persistence of the disease. Created with Figdraw.com.

As shown in Figure 2, building upon the inflammatory microenvironment established by a robust innate immune response, the pathological progression of GM enters a critical phase dominated by adaptive immunity. T lymphocytes and B lymphocytes are activated and extensively recruited to the lesion sites (52). Meanwhile, HPRL, as a significant immunomodulatory factor, is deeply involved in and exacerbates the immune imbalance at this stage (53). The hallmark of this phase is a marked dysregulation of T-cell and B-cell function, which ultimately leads to the formation of the characteristic non-caseating granulomatous structures (35). T cells represent the predominant population of infiltrating lymphocytes within GM lesions. Unlike classical Th1-dominant granulomatous diseases, evidence indicates that in both local lesions and patient peripheral blood, levels of Th2-associated cytokines such as IL-4 and IL-6 are elevated, while levels of Th1-associated cytokines such as IL-2 and IFN-*γ* are relatively reduced, suggesting a polarization of the immune response toward a Th2 phenotype (54). Immunologically, IL-4 is the key cytokine driving the differentiation of naive T cells into the Th2 lineage (55). In the context of GM, it is particularly noteworthy that the recently described pathogenic Th2 cells, upon stimulation with IL-33, can highly express IL-5 (56, 57). This may exacerbate the eosinophilic inflammation observed in GM (58).

Concurrently, the Th17 cell response is aberrantly augmented in GM. Within a microenvironment shaped by factors such as IL-6 and TGF-*β*, Th17 cells differentiate and secrete IL-17 (54, 59). Studies have shown that serum IL-17 levels are significantly elevated in the peripheral blood of GM patients (60). As a potent neutrophil chemokine, the overproduction of IL-17 may contribute to the neutrophil infiltration and microabscess formation observed in GM lesions (61, 62). Based on the above findings, we hypothesize that the Th17 response is a key mechanism underlying the suppurative destructive features of GM. Notably, the Th1 response, which mediates cellular immunity, may not be the predominant driver in GM. Intriguingly, some studies have found that ozone therapy, by enhancing the secretion of Th1-type cytokines such as IFN-*γ* and TNF-α, can contribute to the alleviation of clinical symptoms. This suggests that within the specific microenvironment of GM, Th1 function may be in a state of relative imbalance (63). Furthermore, cytotoxic T lymphocytes are extensively infiltrated into lesions during the active stage of GM, potentially exacerbating tissue damage through direct cytolytic effects and/or cytokine secretion (52).

#### 4.3.2 Abnormal activation of B lymphocytes

Current research indicates that HPRL can disrupt B-cell immune tolerance in a variety of autoimmune diseases. The underlying mechanisms include enhancing the survival and proliferation of mature B cells and, through synergy with B-cell receptor signaling, lowering their activation threshold. Consequently, autoreactive B cells become more prone to escaping clonal deletion or anergy, leading to their persistent presence and aberrant activation in the periphery (64, 65). Unfortunately, current research has not elucidated the precise mechanisms by which HPRL contributes to the development of GM. Further investigation in this area warrants future exploration by researchers.

Upon activation and with help from Th cells, B cells differentiate into plasma cells, which secrete immunoglobulins. This leads to generally elevated levels of IgA, IgG, and IgM in both the pus and serum of GM patients (35). Among these immunoglobulins, IgG4, an antibody subclass associated with Th2-type responses, is of particular interest (66). Studies have shown a significant increase in IgG4 + plasma cell infiltration within lesions, especially in GM patients with anatomical factors such as nipple retraction. This suggests a potential link between IgG4 and the processes of ductal obstruction, ductal endothelial cell injury, and nipple retraction (67).

Activated B cells and plasma cells serve not only as antibody producers but also as important sources of the pro-inflammatory cytokines IL-6 and TNF-α. Studies have demonstrated significantly elevated levels of both IL-6 and TNF-α in the lesional tissue of GM patients (68, 69). Based on the integrated evidence, we hypothesize that these factors may act in concert with PRL to form a core positive feedback loop that drives the chronicity of inflammation: PRL can promote the secretion of factors such as IL-6 and IFN-*γ* by immune cells, driving the polarization of the immune microenvironment towards a pro-inflammatory state (70). IL-6 derived from B cells and other PRL-influenced cells serves as a key factor promoting Th17 cell differentiation. In turn, activated Th17 cells and the inflammatory milieu they create may further support the activation of immune cells (71–73). Simultaneously, TNF-α potently recruits neutrophils and macrophages (74, 75). The inflammatory mediators released by these cells, combined with their sustained antigen-presenting function, further stimulate the activation of T and B cells, thereby enabling the self-perpetuation of the local inflammatory microenvironment. However, the detailed molecular interactions within this proposed feedback loop require further experimental validation (68, 69).

#### 4.3.3 Structural formation of granulomas

Macrophages serve as the central architects of granulomatous structures. Upon activation, they can polarize into distinct functional phenotypes. In GM, however, findings regarding the specific phenotypes of macrophages have been divergent. Some studies suggest that the epithelioid macrophages forming the granuloma core in GM exhibit a pro-inflammatory M1 phenotype (23, 76); while others indicate that CD68- and CD163-positive M2 macrophages predominate in the lesions (77). This discrepancy may reflect the microenvironmental heterogeneity within different regions of GM granulomas. Within GM lesions, aggregated epithelioid macrophages and multinucleated giant cells formed by macrophage fusion can be observed (78). In classical granuloma models, this fusion process is typically driven by sustained stimulation from cytokines such as TNF-α. Given the significant elevation of TNF-α within GM lesions, a similar driving mechanism is likely at play in this disease (23, 79). Beyond cellular immunity, humoral immunity is also deeply involved: a significantly greater infiltration of plasma cells is observed in the lesions compared to infectious granulomas, supporting the involvement of an autoimmune mechanism (80); Systemic immune dysregulation is also evident in the peripheral blood of patients, characterized by elevated proportions of cytotoxic T lymphocytes, NK cells, and NKT cells. Notably, NKT cells have been shown in models to regulate granuloma formation (81, 82). Ultimately, driven by the chronic inflammatory microenvironment which is characterized by the aforementioned cytokine alterations and immune cell infiltration, these cellular components including epithelioid cells, multinucleated giant cells, lymphocytes, and plasma cells assemble in an organized manner and interact with one another, finally giving rise to the characteristic pathological structure of a non-caseating granuloma (23).

### 4.4 Imbalance stage: Treg/Breg defect and disease chronification

Treg cells are essential for maintaining immune tolerance. However, during the active stage of GM, both their numbers and function are impaired. Studies have confirmed that GM patients exhibit a reduced population of Treg cells in peripheral blood, accompanied by significantly decreased expression of FOXP3, the key transcription factor essential for their identity and suppressive function (73, 83). This deficit directly results in a relative predominance of effector T cells, leading to an imbalance in the Teff/Treg ratio. Consequently, the immune system fails to adequately restrain the overactive inflammatory response (35). Notably, HPRL may further compromise Treg function. PRL binds to receptors on the Treg cell surface, interfering with their suppressive activity. This indirectly fuels the proliferation of effector cells and the secretion of the pro-inflammatory cytokine IFN-*γ*, thereby creating a vicious cycle (73). Based on current evidence regarding Th17 cells and GM, it is reasonable to hypothesize that an imbalance between Th17 and Treg cells exists in GM patients, which hinders the effective resolution of inflammation.

Breg cells represent another crucial class of immunoregulatory cells (84). Their functional impairment in GM, coupled with the aforementioned Treg cell deficiency, constitutes a dual failure of immunoregulation. Breg cells, particularly the subset that exerts its function via IL-10 secretion, are essential for maintaining immune tolerance and homeostasis. They directly suppress the activity of various pro-inflammatory immune cells, including Th1, Th17, and CD8 + T cells, as well as NK cells and dendritic cells. Furthermore, they can induce the differentiation of naive CD4 + T cells into Treg cells, thereby establishing a critical peripheral immunosuppressive barrier (85). However, this regulatory barrier is markedly compromised in GM patients. Studies reveal that patients in the active stage exhibit a significantly reduced proportion of Breg cells in peripheral blood (73). Correspondingly, serum levels of the key anti-inflammatory cytokine IL-10 are also found to be low in these patients (86). The reduction in Breg cells and the low levels of IL-10 indicate an impaired capacity of this pathway to suppress excessive immune responses. This impairment may allow effector cell reactions, such as those driven by Th17 cells, to proceed unchecked, hindering the self-containment of the inflammatory process. Currently, research on the precise regulatory mechanisms governing Breg cells in GM remains relatively limited. Therefore, exploring how to correct Breg cell deficiency and restore IL-10-mediated immunoregulatory function represents a promising direction for future research into GM treatment mechanisms.

This immune imbalance in GM is not confined to the active stage alone. A longitudinal study revealed that, even during the asymptomatic remission phase when lesions had subsided, the expression level of Foxp3, the master transcription factor for Treg cell function, remained significantly lower in patients’ peripheral blood compared to healthy controls (73). Another study similarly reported that the serum levels of soluble triggering receptor expressed on myeloid cells-1, a biomarker reflecting the activation state of myeloid cells such as macrophages and neutrophils, remained persistently higher in patients during remission compared to healthy controls (87). These findings collectively suggest that the “clinical remission” of GM may signify only a temporary quiescence of local inflammation, while the underlying systemic immune dysregulation does not resolve. This persistent systemic immune abnormality likely explains the high risk of recurrence even after clinical remission is achieved.

## 5 Comparative analysis of GM and systemic autoimmune disease features

The immune dysregulation observed in GM exhibits distinct systemic characteristics. The evidence supporting its autoimmune nature is multifaceted and mutually reinforcing. First, its pathogenesis is primarily immune-mediated, as evidenced by granuloma formation, responsiveness to GC therapy, and frequent association with systemic immune phenomena such as erythema nodosum and arthritis (88). Second, potential pathways driving systemic responses exist, such as perinatal hormonal fluctuations and elevated prolactin levels. These may simultaneously perturb both mammary epithelial cells and the systemic immune system, likely via signaling pathways like JAK/STAT, thereby initiating an inflammatory cascade that progresses from a local to a systemic scale (76, 89). Third, GM patients frequently exhibit comorbidity with other autoimmune diseases, such as systemic lupus erythematosus (SLE) and Sjögren’s syndrome, reflecting a shared background of polyautoimmunity (23). Finally, its dependence on immunosuppressive treatment, as well as its sensitivity to seasonal fluctuations, aligns with the pattern observed in classical autoimmune diseases (23).

These systemic characteristics of GM bear a striking resemblance to those of classical systemic autoimmune diseases, such as SLE. At the cellular level, lesions in both conditions exhibit a T-lymphocyte-dominated infiltration pattern (90). Regarding humoral immunity, both demonstrate a clear autoimmune humoral response. Antinuclear antibodies (ANA), a hallmark of systemic autoimmunity, are detected in a considerable proportion of GM patients. Notably, even antibodies with high diagnostic specificity for SLE, such as anti-double-stranded DNA antibodies, can be present in the serum of some GM patients (91, 92). More compellingly, studies applying SLE classification criteria for evaluation have found that approximately 20% of GM patients achieve a positive score. This observation provides clinical standard-based evidence for an overlap in autoimmune manifestations between the two conditions (93). Furthermore, studies indicate that the HLA-DRB1*17 allele is found in both GM and SLE patients, suggesting a shared component of genetic susceptibility between the two diseases (94).

In summary, GM not only exhibits multiple lines of evidence characteristic of systemic autoimmune diseases but also shows significant overlap with SLE across several dimensions, including cellular infiltration patterns, autoantibody profiles, clinical classification criteria, and genetic background. Consequently, GM may warrant being reconsidered and reclassified as a systemic autoimmune disorder, rather than a purely localized inflammatory condition. This reconceptualization may help explain its persistent clinical challenges, including resistance to cure and a tendency toward chronicity and recurrence. Furthermore, it provides a rationale for exploring treatment strategies borrowed from systemic autoimmune diseases.

## 6 Clinical presentation and disease course

The main manifestation of GM is a palpable breast lump, which may or may not be accompanied by pain and can increase rapidly in size over a short period of time. This condition is often accompanied by localized breast inflammation, including engorgement, swelling, warmth and pain, as well as nipple discharge and orange peel-like skin changes (95). Typical masses are firm in texture, irregular in shape with indistinct borders, and often adhere to adjacent tissues. A retrospective study by Martinez-Ramos et al. (21) involving 3,060 patients confirmed that 80% of cases presented with breast masses averaging 5 cm in size (range 3–9 cm), with 66% experiencing pain. As GM progresses, breast lumps can develop into abscesses that fluctuate on palpation (2). Sinus tracts, skin ulcers, or wounds that are prolonged may occur in advanced stages of the disease. Patients may present with systemic symptoms such as low-grade fever and general malaise, in addition to axillary lymph node enlargement. In addition, systemic manifestations such as erythema nodosum and arthritis may occur at any stage of GM, while corneal limbitis is rare (96, 97). Among 474 GM patients studied by Azizi et al. (98), 15.6% presented with nipple discharge, and 4.6% reported joint pain. A review by Li et al. (99) of 201 GM patients reported the following extra-mammary and local manifestations: erythema nodosum in 4.5%, papilledema in 7.5%, and sinus tracts in 49.8% of cases. The study also found that sinus tract formation was more common and statistically significant in patients with *Clostridium difficile* infection, suggesting a potential link between sinus tract formation and this infection.

## 7 Diagnosis and differential diagnosis

Clinical diagnosis and histopathological features are essential for diagnosing GM. The diagnostic process typically includes clinical presentation, imaging, laboratory tests to rule out other potential breast diseases such as autoimmune disorders, as detailed in Table 1, and biopsy for histological examination of the tissue. However, there are currently no internationally standardized guidelines for the diagnosis and treatment of GM (2).

**Table 1:** TCM syndrome differentiation, treatment principles, representative formulas.

TCM syndrome pattern	Treatment principle	Representative formula	References
Liver Qi Stagnation with Phlegm Coagulation Pattern	Soothing the liver and regulating qi, resolving phlegm and dissipating nodules	Chaihu Qinggan Decoction	(131, 132)
Intense Heat Toxin Pattern	Clearing heat and draining fire, cooling blood and resolving toxin	Heat-Clearing and Toxin-Resolving Formula	(131, 133)
Yang Deficiency with Toxin Accumulation Pattern	Warming Yang and freeing stagnation	Yanghe Decoction	(131, 134–136)

### 7.1 Laboratory tests

Common laboratory tests include a complete blood count, erythrocyte sedimentation rate, purified protein derivative testing, and inflammatory markers such as C-reactive protein and serum PRL. Immunologic tests may include an antinuclear antibody profile and rheumatoid factor.

First, complete blood count is typically not significantly abnormal in the early stages of GM. However, as the disease progresses, especially during the abscess formation stage, the white blood cell count may increase, indicating an acute inflammatory response (100). erythrocyte sedimentation rate is a nonspecific indicator of inflammatory activity in the body. During the acute or abscess phase of GM, some patients may show an increased erythrocyte sedimentation rate, which suggests ongoing inflammation (101). Although the purified protein derivative test has little value in diagnosing GM itself, it is still helpful when TM needs to be ruled out (2).

.Inflammatory markers are helpful for assessing how active GM is. For instance, C-reactive protein, a common acute-phase protein, often rises during flares or if infection is present. Shifts in C-reactive protein levels can help gauge how severe the inflammation is. Some GM patients also show higher serum PRL, especially when breast tissue is damaged or inflamed. Therefore, if PRL is high, doctors usually recommend checking it again later to monitor any changes over time (100).

Immunological tests are used to rule out other immune disorders in the diagnostic process for GM. Therefore, if signs like erythema nodosum appear, it suggests possible immune-related mechanisms, as some GM patients do exhibit irregularities in their immune function (102, 103). In these situations, antinuclear antibody testing is useful to detect systemic lupus erythematosus or other connective tissue diseases. For GM patients with joint symptoms, rheumatoid factor testing helps exclude rheumatoid arthritis (91). Measuring immunoglobulins and complement proteins is also important for evaluating immune function and detecting possible immune issues, particularly in GM patients with systemic conditions or immune dysregulation (49, 103).

### 7.2 Breast ultrasound

**Figure 3:**
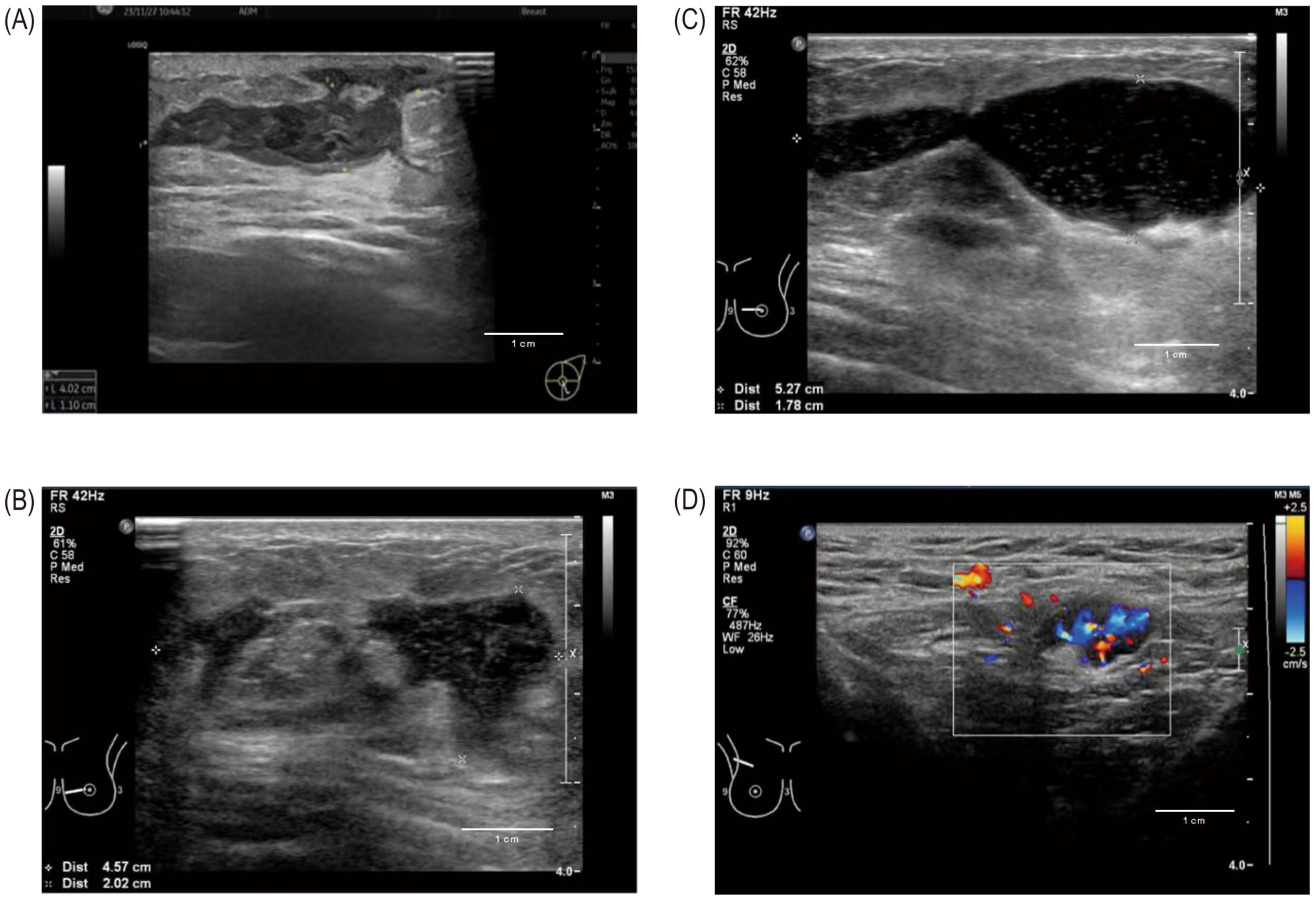
Typical ultrasound appearances in GM. **(A)** A central lesion showing an irregular, hypoechoic mass. **(B)** Multiple localized abscesses appearing as fluid-filled areas. **(C)** A fully developed abscess displaying a clear, fluid-filled space with enhanced through-transmission. **(D)** An enlarged axillary lymph node which shows a preserved fatty hilum and increased blood flow at its center.

Breast ultrasound (USG) serves as the primary imaging technique for patients suspected of having GM. It clearly visualizes the lesion and can determine the number, size, and position of any abscesses. Common ultrasound features include irregular hypoechoic masses (Figure 3A) and multiple fluid-filled abscesses (Figures 3B,C). These findings are often accompanied by skin thickening, subcutaneous edema, and axillary lymphadenopathy (Figure 3D). Tubular hypoechoic areas on ultrasound may suggest duct involvement or fistula formation. Concurrently, color Doppler imaging frequently demonstrates increased blood flow around abscesses, aiding in the assessment of inflammatory activity and severity (104).

### 7.3 Enhanced magnetic resonance imaging

Magnetic resonance imaging (MRI) plays a useful role in both diagnosing and monitoring GM. MRI provides excellent soft tissue resolution, making it a key tool for complex cases. This version uses “provides clear pictures” and “how well treatment is working” for simpler expression, and connects the two sentences with “Because of this capability” for natural flow. MRI provides clear pictures that help doctors evaluate a lesion’s size, level of inflammation, and how well treatment is working. Because of this capability, it is especially valuable when ultrasound findings are inconclusive or when more precise details about the lesion are needed (105).

On MRI, GM most commonly presents as non-mass enhancement. This pattern frequently occurs in a segmental, ductal, or regional distribution, suggesting inflammatory changes. When a mass is present, it typically exhibits irregular borders and marginal enhancement, potentially indicating abscess formation or necrotic tissue (106). On T1-weighted images, these lesions usually appear as low signal intensity. On T2-weighted fat-suppressed sequences, lesions appear hyperintense, reflecting their fluid-rich, inflammatory nature. Although most GM lesions exhibit a Type I time-signal curve, a minority demonstrate a Type III curve. This Type III pattern overlaps with the typical curve seen in breast cancer (107). Diffusion-weighted imaging frequently demonstrates diffusion restriction in GM lesions, manifested as low apparent diffusion coefficient values—a feature also observed in inflammatory breast cancer (IBC). Accurate diagnosis requires integrating these MRI findings with clinical presentation and other imaging results for comprehensive interpretation (108). In the management of GM, MRI serves several important purposes. Firstly, it helps determine the exact location and extent of lesions. It also monitors how the disease changes throughout treatment. Furthermore, MRI assesses long-term treatment effectiveness, offering essential imaging support across the entire care process (105).

### 7.4 Pathological findings

**Figure 4:**
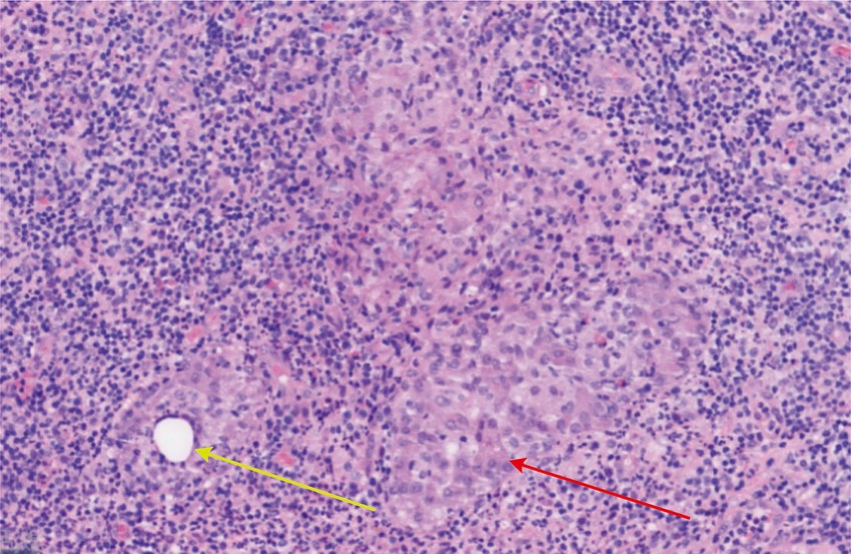
GM in idiopathic granulomatous mastitis (H&E staining; 200×). Non-caseating granulomas (red arrowheads), comprised of epithelioid histiocytes and multinucleated giant cells, and scattered microabscesses (yellow arrows) are evident within the breast lobule.

**Figure 5:**
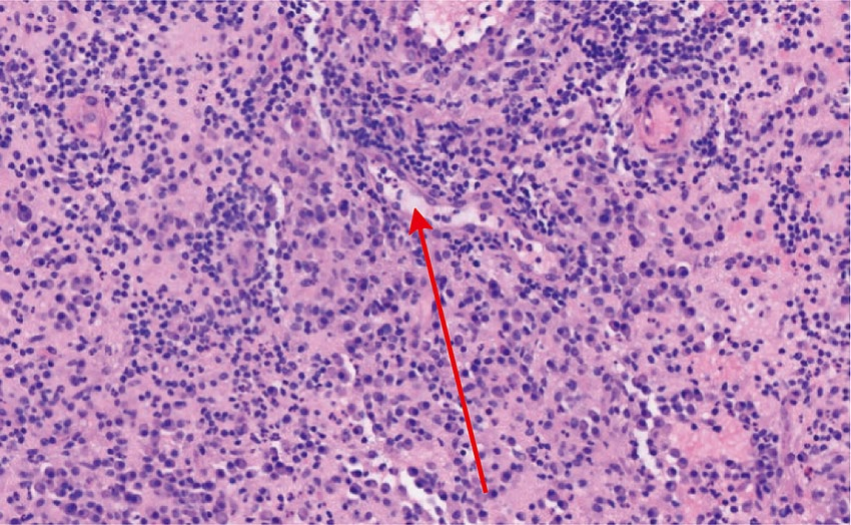
Histological appearance of the acute suppurative phase in GM (H&E staining; 200×). A large number of neutrophils are observed within the vascular lumen (indicated by red arrowheads), surrounded by an infiltrate of plasma cells, epithelioid histiocytes, foamy macrophages, and lymphocytes.

In its symptoms and imaging features, GM can look very similar to a common breast abscess or to cancers like IBC. Because of this similarity, a definite diagnosis cannot be made on clinical and radiological findings alone and usually requires a core needle biopsy for tissue analysis (98). The key pathological feature of GM is non-caseating granulomatous inflammation centered on breast lobules. Lesions are often distributed in multiple foci, which can partially or completely destroy the normal lobular architecture. The inflammatory infiltrate consists of neutrophils, lymphocytes, plasma cells, eosinophils, and epithelioid histiocytes. These cells gather to form granulomas within the affected area, sometimes accompanied by sterile microabscesses, as shown in Figures 4, 5 (23, 95). Some cases also show dilation of ducts within and between lobules, along with fat necrosis and fibrosis. These features are important for diagnosing GM and distinguishing it from other conditions (23).

## 8 Management and therapeutic strategies

### 8.1 Conventional therapeutic strategies for GM

Therapeutically, GM presents a distinct clinical challenge. As an idiopathic granulomatous inflammation of unknown etiology, its management remains controversial in clinical practice. Commonly employed treatment modalities include observation, oral antibiotics, glucocorticoids (GCs), surgical excision, and oral traditional Chinese medicine therapy (6). GM is considered to have a self-limiting nature. According to the literature, approximately 50% of GM patients may experience a natural remission period ranging from 2 to 24 months (2). Studies have shown that for localized lesions that are small, unilateral, and have not formed abscesses or chronic sinus tracts, some patients can undergo spontaneous resolution without pharmacological or surgical intervention (109). Therefore, for patients with mild, localized clinical symptoms that show no signs of rapid progression, a conservative strategy of regular clinical surveillance is justifiable after thorough patient-clinician communication. However, a meta-analysis incorporating 4,735 patients indicated that the recurrence rate under a simple watchful waiting approach was significantly higher compared to other active interventions (110).

In the therapeutic management of GM, antibiotic use should be reserved for cases with clear indications and requires prudent consideration. This recommendation primarily stems from the possible concomitant bacterial infection observed in some cases, with Corynebacterium being a notable example (111). A retrospective study utilizing clarithromycin as first-line therapy reported an average time to clinical remission of approximately 8 months, suggesting its potential efficacy in specific scenarios. However, the therapeutic benefit is significantly limited, as antibiotic monotherapy often proves insufficient to adequately control the disease course. The same study found that nearly one-third of patients experienced recurrence during follow-up, and almost half required combination therapy with GCs to effectively manage inflammation (112). Consequently, current consensus emphasizes that once GM is diagnosed, empirical long-term antibiotic therapy without clear indications should be avoided (104).

GCs currently represent an effective therapeutic approach for GM (113). Clinical studies have confirmed that approximately 80% of GM cases respond effectively to GC treatment, making GCs a first-line option for moderate to severe or progressive GM (114). However, there is no standardized protocol for the clinical use of GCs. The typical initial dose ranges from 0.5 to 1 mg/kg/day in prednisone equivalent, maintained for several weeks, and is then tapered gradually based on the treatment response (114, 115). This systemic administration can achieve complete remission in most patients, although the treatment duration is prolonged, with a median time to remission reaching 159 days in one study (116). GC therapy is confronted with two major challenges: a high recurrence rate and significant side effects. Approximately 50% of responders experience relapse within 1–2 months after discontinuation, with some patients even requiring a second treatment course (114). Furthermore, long-term or high-dose use can lead to adverse effects such as Cushing’s syndrome, weight gain, and hyperglycemia (2, 117).

While traditional oral systemic administration remains the mainstay of treatment, topical application of GCs is emerging as an alternative approach that reduces systemic side effects and is gaining recognition (114, 118). Studies indicate that topical therapy can induce a clinical response in over 80% of patients within 12 weeks, with associated lower rates of recurrence and side effects (118).

Prior to the use of GCs for GM management, surgical excision was the primary therapeutic approach (5). Surgical options included procedures such as wide local excision, partial mastectomy, and total mastectomy, with the goal of completely removing the involved inflammatory tissue (8). For cases complicated by abscess formation, ultrasound-guided needle aspiration or surgical incision and drainage are essential interventions (119). However, surgical intervention carries significant limitations. The foremost concern is the high recurrence rate. According to the literature, the overall recurrence rate of GM ranges from 5 to 50%, whereas recurrence following surgical excision can be as high as 50% (13). Furthermore, surgery may lead to a range of complications, including delayed wound healing, poor cosmetic outcome, mammary fistula formation, nipple retraction, skin flap necrosis, hematoma, and chronic pain (120). Given these limitations, surgery is not considered an essential or first-line treatment for GM. It is primarily indicated for the following scenarios: 1 failure of pharmacological therapy; 2 contraindications to drug treatment, such as pregnancy; and 3 localized abscesses that have failed needle aspiration drainage (109, 117, 121). For the rare, refractory cases in which all other medical approaches have been exhausted, mastectomy may be considered as a last resort (114).

For GM patients who have not responded to antibiotics, GCs, or surgery, immunosuppressants serve as an important subsequent treatment option. Among these, methotrexate is the most widely used agent, typically employed as second-line therapy or in combination with GCs (114). Studies have demonstrated that for cases refractory to antibiotics, GCs, and surgery, treatment with methotrexate achieved improvement in 94% of patients and clinical remission in 75% after a 15-month course (15). When an methotrexate and GC combination regimen is employed, case series reports indicate that complete symptom resolution can be achieved in 72 to 100% of patients, typically within 2 to 4 months of treatment (114). However, potential side effects, such as elevated liver enzymes and alopecia, necessitate its use under the collaborative management of rheumatology and breast specialists (122).

### 8.2 Emerging therapeutic strategies for GM

While conventional treatments for GM often face challenges such as high recurrence rates and drug-related side effects, recent years have witnessed the emergence of novel therapeutic approaches targeting its immunopathological mechanisms, providing clinicians with expanded options.

For patients with refractory or severe GM who respond poorly to conventional therapies, biologic agents targeting TNF-α have shown promise. In one study, a patient who failed intensive GC and methotrexate therapy demonstrated marked improvement after treatment with the TNF-α inhibitor adalimumab (123). More compelling evidence comes from a well-documented case involving a GM patient with concurrent psoriasis. The patient’s symptoms resolved with adalimumab, recurred upon discontinuation, and rapidly improved again within 1 week of re-initiating the treatment, providing robust evidence for the drug’s direct therapeutic effect (124). These case reports suggest that TNF-α inhibitors may serve as a potential effective alternative to surgery and non-systemic GC therapy.

By blocking the JAK–STAT signaling pathway, JAK inhibitors broadly suppress the inflammatory response at its upstream source, offering a novel oral targeted therapeutic strategy for GM (23, 71). Although direct clinical data on JAK inhibitors for GM are still accumulating, their mechanism of action is highly relevant to the immunological features of GM. This relevance is supported by the established efficacy of JAK inhibitors in other conditions such as rheumatoid arthritis. Therefore, they offer a novel conceptual direction for GM treatment (23, 125, 126).

Intraductal lavage therapy represents an innovative local treatment, which involves pumping medicated irrigating fluid into the affected ducts for lavage. A prospective study demonstrated that this approach achieved a clinical complete remission rate exceeding 90% at 1 year, with no significant adverse events reported (127). This suggests that local intraductal lavage therapy may represent one of the future directions for therapeutic optimization.

Intralesional steroid injection involves the direct administration of GCs into granulomatous lesions, thereby achieving a potent localized anti-inflammatory effect while minimizing systemic exposure and associated side effects (124, 128). A prospective controlled study confirmed that patients receiving intralesional injection combined with topical steroid application demonstrated a significantly higher response rate at 3 months and a notably lower recurrence rate compared to those on oral steroids alone. Moreover, systemic side effects were predominantly observed in the oral steroid group (128).

### 8.3 Application of TCM in the management of GM

While GCs and immunosuppressants form the cornerstone of conventional Western medical therapy for GM, their long-term administration is limited by associated side effects in clinical practice (2). Against this backdrop, Traditional Chinese Medicine (TCM), which emphasizes holistic regulation, has gained increasing attention as a complementary therapeutic approach. TCM treatment is not merely a list of herbal formulas but constitutes a systematic therapeutic strategy guided by its own theoretical framework. Based on clinical practice, different TCM practitioners hold varying interpretations regarding the pathogenesis of GM. For instance, Professor Lin Yi classifies GM within the TCM disease category of “sores and ulcers”, considering it predominantly a “Yin syndrome.” He emphasizes that internal treatment in the later stages should primarily focus on fortifying the spleen, replenishing qi, and harmonizing the nutrient aspect, aiming to promote wound healing and shorten the disease course (129). Professor Lou Lihua posits that the disease fundamentally involves Yang Qi deficiency with cold congelation and phlegm stagnation. The treatment should therefore follow the principles of warming Yang and dissipating cold, resolving phlegm and dispersing stagnation, as well as nourishing blood and freeing stagnation. The formula Yanghe Decoction, modified according to presentation, is applied. This approach is suitable for patients presenting with breast masses of a deficiency-cold nature (130).

Here, we adopt the framework outlined in the Beijing Expert Consensus on the Integrated Traditional Chinese and Western Medicine Diagnosis and Treatment of Granulomatous Mastitis (2025 Edition), classifying GM into three fundamental patterns for pattern-based treatment: Liver Qi Stagnation with Phlegm Coagulation Pattern, Intense Heat Toxin Pattern, and Yang Deficiency with Toxin Accumulation Pattern (131). The Liver Qi Stagnation with Phlegm Coagulation Pattern predominantly manifests in the early stages of the disease. It is treated by soothing the liver and regulating qi, resolving phlegm and dissipating nodules, with a representative formula being the classical formula Chaihu Qinggan Tang from The Orthodox Lineage of External Medicine (131, 132). Studies have demonstrated that the combination of Chaihu Qinggan Decoction with prednisone can modulate immune function. This is specifically manifested as an increase in peripheral blood levels of CD3 + and CD4 + T cells and the CD4+/CD8 + ratio, alongside a reduction in immunoglobulin levels such as IgG, IgA, and IgM. These findings suggest that the therapeutic effect may be mediated by restoring the balance of T lymphocyte subsets and suppressing excessive humoral immunity (132). The Intense Heat Toxin Pattern corresponds to the stage of acute inflammation or abscess formation. The treatment principle focuses on clearing heat and draining fire, cooling blood and resolving toxin, typically using modified formulations based on the Heat-Clearing and Toxin-Resolving Formula (131). Research indicates that the Heat-Clearing and Toxin-Resolving Formula not only significantly reduces the area of breast redness and swelling as well as the size of masses, but also decreases serum levels of white blood cell count, neutrophil count, and C-reactive protein, demonstrating both anti-inflammatory and immune-modulating effects. Modern pharmacological studies confirm that core herbal components of the formula, such as *Forsythia suspensa* and Taraxacum mongolicum, may alleviate the inflammatory response by inhibiting signaling pathways including NF-κB, thereby downregulating the expression of pro-inflammatory cytokines such as IL-6, IL-1β, and TNF-α (133). The Yang Deficiency with Toxin Accumulation Pattern is commonly seen in a state of deficiency and lingering illness, characterized by protracted disease course and non-healing wounds. Yanghe Decoction, as a representative formula for warming Yang and freeing stagnation, can improve the local microenvironment of yin-cold congelation and stagnation, thereby promoting tissue repair and wound healing (131). Zhang et al. (134) found that Yanghe Decoction combined with surgical intervention demonstrated a higher cure rate and a lower recurrence rate compared to surgery alone, suggesting its therapeutic potential in the management of GM. Moreover, modern research has found that Modified Yanghe Decoction can downregulate the expression of pyroptosis-related proteins, such as NLRP3 and Caspase-1, in GM lesions. Given that the pyroptosis pathway has been shown to be activated in GM lesions, this finding suggests that the herbal formula may exert its therapeutic effect by modulating this inflammatory cell death pathway (135, 136).

For GM cases that have progressed to abscess formation or ulceration, external treatment methods such as Tounong San irrigation and drainage or external application of Jinhuang Gao can be employed to promote pus discharge, reduce swelling, and alleviate pain (86, 137). A clinical study demonstrated that the combined use of Chinese herbal decoctions with external application of Jinhuang Gao effectively increased the proportion of peripheral blood Treg cells and the levels of IL-10 and TGF-β1, thereby regulating the Treg/Th17 immune balance. This regimen also showed favorable safety and a lower recurrence rate (86). Additionally, therapies such as fire-needle cupping and pricking-cupping can clear meridians and drain heat-toxins through physical stimulation (35). A large-scale clinical observation demonstrated that the therapeutic approach of “Regulating Qi and Harmonizing the Nutrient System” combined with fire-needle cupping achieved a total effective rate of 86.38% in treating GM. This regimen significantly increased the levels of peripheral blood CD3+, CD4+, and CD8 + T cells, while reducing the levels of NK cells, IgM, and complement components C3/C4, thereby improving clinical symptoms and comprehensively modulating immune function (138).

TCM treatment alone offers advantages such as a favorable safety profile and preservation of breast aesthetics, but it typically requires a relatively long treatment duration (35, 139). Therefore, integrated traditional Chinese and Western medicine has become a key strategy in the current clinical management of GM. For instance, a study employing a combination of Chinese herbal medicine and surgical excision with suture reported that this regimen significantly improved the cure rate, shortened the treatment course, and reduced the rate of lesion suppuration (140). Furthermore, the combination of Western pharmaceuticals with targeted Chinese herbal penetration therapy has also demonstrated advantages in enhancing the overall response rate, reducing recurrence, alleviating symptoms, and preserving breast aesthetics (141).

In summary, through the principles of pattern differentiation and multi-pathway intervention, TCM demonstrates significant potential in the treatment of GM by enhancing therapeutic efficacy, reducing recurrence rates, and mitigating the side effects associated with conventional Western medications. Nevertheless, the current body of evidence predominantly originates from small-sample, retrospective studies, and the level of evidence requires further elevation. Future efforts should prioritize well-designed prospective randomized controlled trials to generate higher-level evidence, coupled with in-depth pharmacological research to elucidate the underlying mechanisms of action (142).

## 9 Conclusion

In conclusion, GM is a chronic disease with complex etiology and variable manifestations, which has been a clinical challenge due to the lack of standardized diagnostic and therapeutic criteria. In this article, we analyzed all aspects of the disease and found that GM is not only a local inflammation of the breast, but also a systemic autoimmune disorder, in which the patient’s innate and adaptive immune systems are “overreacting,” and a variety of immune cells, such as macrophages and lymphocytes, are involved. Hyperprolactinemia is an important risk factor for exacerbating this problem. Therapeutically, although GCs are commonly used, they are prone to recurrence and side effects; surgical interventions are also becoming more and more cautious, and the overall direction of treatment tends to be conservative. Against this backdrop, complementary therapies such as TCM, with its unique role in regulating immunity and preventing recurrence, offer new hope for the comprehensive treatment of GM.

Despite significant progress in understanding GM, core elements of its etiology and pathogenesis remain to be fully elucidated, and standardization of diagnosis and treatment is urgently needed. Future studies should focus on the following key directions: at the mechanistic level, there is a need to integrate multi-omics techniques to deeply analyze the genetic susceptibility of GM, the interactions between the local immune microenvironment and microorganisms (97), and to focus on elucidating the molecular mechanisms that drive specific autoimmune responses, including the specific roles of NETs, specific T-cell subsets, and IgG4-positive plasma cells in the pathologic process (2, 67). At the clinical level, efforts should be made to develop non-invasive diagnostic tools based on serum biomarkers, radiomics or liquid biopsies, and to optimize the assessment using artificial intelligence for early and differential diagnosis (2). From a therapeutic perspective, there is a need for large-scale prospective clinical trials to validate the efficacy and safety of different therapeutic options (11, 143), to promote the development of personalized therapeutic strategies, and to scientifically validate traditional medical therapies, including TCM (2, 144).

Therefore, improving the long-term prognosis and quality of life of patients with GM ultimately requires the mutual integration of basic research and clinics and continuous innovation in diagnosis, treatment, and guideline development.
